# Natural COA water inhibits mitochondrial ROS-mediated apoptosis through Plk3 downregulation under STZ diabetic stress in pancreatic β-cell lines

**DOI:** 10.1016/j.bbrep.2022.101247

**Published:** 2022-03-11

**Authors:** Jeyeon Lee, Jin Ook Chung, Seon-Young Park, Naveen Rajamohan, Aparna Singh, JungJin Kim, Val J. Lowe, SeungBaek Lee

**Affiliations:** a Division of Radiology, Mayo Clinic, Rochester, MN, 55905, USA; b Department of Internal Medicine, Chonnam National University Medical School, Gwangju, 501757, Republic of Korea; c Department of Molecular Pharmacology and Experimental Therapeutics, Mayo Clinic, Rochester, MN, 55905, USA

**Keywords:** Diabetes, Pancreatic β-cells, Reactive oxygen species (ROS), Natural COA water, Plk3, Apoptosis, 3D organoid, *ex vivo*, ROS, reactive oxygen species, COA, Charisma of Aqua, STZ, Streptozotocin, Plk3, Polo-like kinase 3, 3D, three-dimensional, APC, anaphase-promoting complex, MAVS, mitochondrial antiviral signaling protein, DKD, diabetic kidney disease, ΔΨm, mitochondrial membrane potential, AQP, aquaporin, MMP, mitochondrial membrane potential

## Abstract

Diabetes from pancreatic β cell death and insulin resistance is a serious metabolic disease in the world. Although the overproduction of mitochondrial reactive oxygen species (ROS) plays an important role in the pathogenesis of diabetes, its specific molecular mechanism remains unclear. Here, we show that the natural Charisma of Aqua (COA) water plays a role in Streptozotocin (STZ) diabetic stress-induced cell death inhibition. STZ induces mitochondrial ROS by increasing Polo-like kinase 3 (Plk3), a major mitotic regulator, in both Beta TC-6 and Beta TC-tet mouse islet cells and leads to apoptosis. Overexpression of Plk3 regulates an increase in mitochondrial ROS as well as cell death, also these events were inhibited by *Plk3* gene knockdown in STZ diabetic stimulated-Beta TC-6 cells. Interestingly, we found that natural COA water blocks mitochondrial ROS generation through the reduction of Plk3 and prevents apoptosis in STZ-treated beta cells. Furthermore, using the 3D organoid (*ex vivo*) system, we confirmed that the insulin secretion of the supernatant medium under STZ treated pancreatic β-cells is protected by the natural COA water. These findings demonstrate that the natural water COA has a beneficial role in maintaining β cell function through the inhibition of mitochondrial ROS-mediated cell death, and it might be introduced as a potential insulin stabilizer.

## Highlights

• Pancreatic β cell is stabilized through natural COA water in STZ-induced diabetes.
• Mitochondrial membrane potential (ΔΨm) is controlled by natural COA water.
• Plk3 expression under STZ treatment is negatively regulated by natural COA water.
• Insulin secretion is stabilized by natural COA water under STZ treatment in *ex vivo* (3D organoid) model.

## 1 Introduction

Diabetes mellitus is a disease that occurs when the pancreatic beta cells do not secrete insulin, or the systemic cells are resistant to insulin [[1], [2], [3], [4], [5], [6], [7]]. Diabetes is divided into types 1, 2, and gestational diabetes, a small number of people develop certain types of diabetes due to other causes [1,5,6]. Type 1 diabetes is derived from the loss of normal glucose storage function due to the destruction of the Langerhans beta cells that produce insulin in the pancreas by an autoimmune response. Type 2 diabetes is characterized by chronic hyperglycemia and cellular insulin resistance. Diabetes causes vascular complications related to retinopathy, nephropathy, neuropathy, and cardiovascular disease [6,8]. Gestational diabetes is diabetes that occurs specifically in pregnant women who have not previously been diabetic. This occurs when the body cannot produce enough insulin during pregnancy, and these women are more likely to later develop type 2 diabetes due to insulin resistance. Other types of diabetes include monogenic diabetes syndromes, diseases of the exocrine pancreas, and drug or chemical-induced diabetes [1,5,6]. There are many factors that can lead to diabetes, including mitochondrial dysfunction, increased oxidative stress or Endoplasmic Reticulum (ER) stress, the metabolic problem of mTOR, inflammation, glucotoxicity, and activation of protein kinase C [3,[5], [6], [7],^9^,^10^]. Streptozotocin (STZ) is widely used in research on diabetes in animal models and *in vitro* experiments [1,6,7]. STZ promotes glucose transporter GLUT2 induction, increasing oxidative stress in beta cells, which causes DNA damage and apoptosis via Bax and activated caspase-9 and -3 ^4, 11, 12^. The most ideal treatment for diabetes would be not only effective or long-lasting, but also have minimal side effects to the human body. Although several new drugs are being developed or used in patients, there is still a need to develop an innovative drug that stably secretes insulin and balances the growth of pancreatic beta cells.

Reactive oxygen species (ROS) are highly reactive chemicals that include peroxides, superoxide, hydroxyl radical, singlet oxygen, and alpha-oxygen, formed from O_2_ and are well known as major regulators of important signaling pathways through aerobic metabolism in eukaryotic cells [3,[9], [10], [11],[13], [14], [15], [16], [17]]. ROS are mainly produced in the mitochondria through electron transport chains, peroxisomes by the β-oxidation of fatty acids, and endoplasmic reticulum through the oxidation of proteins [[13], [14], [15], [16], [17]]. Maintaining an appropriate ROS level is vital as it plays an important role in cell growth and survival [13,15,16]. In general, when abnormal ROS are present in cells, redox homeostasis is activated and controlled by antioxidant activity [[13], [14], [15], [16], [17]]. However, in cells that cannot overcome ROS, ROS cause oxidative damage to DNA, proteins, lipids, etc., which leads to organelle damage, apoptosis, and autophagy [1,3,[9], [10], [11],[13], [14], [15], [16], [17]]. Specifically, mitochondrial ROS send damage signals to the cells by disrupting the mitochondrial potential [[13], [14], [15], [16]]. Recently several reports suggested that ROS are strongly related to diabetes [[1], [2], [3], [4],[8], [9], [10], [11]]. In diabetic conditions, chronic hyperglycemia and consequent increase in ROS and cell death exacerbate type 2 diabetes by worsening cellular function and increasing insulin resistance [1,3,4,8,10,11]. Regulators and mechanisms that directly regulate mitochondrial ROS caused by intracellular stress are still not well known.

Polo-like kinases (PLKs) are serine/threonine protein kinases with multiple functions involved in mitosis, meiosis, cytokinesis, DNA replication, chromosome segregation, and various stress response processes [[18], [19], [20]]. Plk members are classified into Plk1, Plk2 (Snk), Plk3 (Fnk, Prk), and Sak (Plk4) in mammals [18]. Plk1 is a mitotic regulator and is localized in the centrosome with another kinase, cyclin B1, Aurora A, etc, during interphase, and phosphorylates and activates components of the anaphase-promoting complex (APC) by regulating the centrosome functions [18,19,21]. Plk1 is overexpressed in several types of human cancers such as lung, breast, and gastric cancer, suggesting that high Plk1 expression is associated with poor prognosis [[18], [19], [20]]. Therefore, it is widely used to study cancer progression and mechanisms. In particular, it is well known that this kinase contributes to the cancer process leading to chromosomal instability. Plk2 and Plk3 are known to act on DNA damage and the G1/S cell cycle, but the mechanism has not yet been specifically reported. Plk4 is known to play a role in the mitotic exit, but the study on its mechanism is insufficient [18]. In a recent study, Plk1 was found in the mitochondria and inhibits IFN induction at the level of mitochondrial antiviral signaling protein (MAVS) through its polo-box domain. Plk1 interacts with MAVS through its Polo-box domain and interferes with the recruitment of TRAF3 to MAVS for the inhibition of IFN. Also, loss of Plk1 induces ROS-mediated autophagy under radiation in breast cancer cells [20]. Plk2 attenuates diabetic kidney disease (DKD)-induced ROS production and mitochondrial membrane potential (MMP) induction and regulates podocyte apoptosis and inflammation [22]. Plk3 expression correlates with galactose-induced diabetic cataract, and loss of Plk3 alleviated galactose-induced cataract formation in a rat model [23]. However, there are insufficient reports on the specific mechanism between diabetes and Plk3.

Water makes up approximately 70% of the human body and has essential functions in the body. The lack of water in the human body brings about various side effects such as dizziness, indigestion, constipation, high fever, depression, and insomnia [[24], [25], [26]]. Therefore, it is important to drink a certain amount of water, but it is also important to check what kind of water you drink. It has been proven by several research papers that high-purity drinking water such as mineral water has a useful function in the human body in various clinical and pharmacological experiments [24,25,[27], [28], [29]]. However, the nature of this mineral water is slightly different due to the purity of the source, the amount of mineral content, and the inclusion of specific trace elements [25]. Furthermore, it has been reported that the pH of the mineral water also has a direct effect on human health [25,26]. Another research paper reported that mineral water increases the activity of natural killer cells for the human immune system and anticancer immunity in mice, or improves atopic dermatitis and lifestyle-related diseases in humans [28]. Additionally, it is reported that certain natural water obtained from Japan not only removes intracellular ROS and suppresses tumor angiogenesis, but also suppresses oxidative damage to pancreatic cells in alloxan-induced type I diabetes model mice [25,27,29]. As such, a growing number of studies are trying to find a link between human health and water [[24], [25], [26], [27], [28], [29], [30]].

In this study we assessed beta-cell function under diabetic stress, such as STZ treatment, by 15 commercially selling drinks of water as a cell incubation source. We found that only the natural COA water stabilized the islet insulin secretion by blocking the mitochondrial ROS caused by STZ treatment. Therefore, in this study, we confirmed that natural COA water had a positive function in preventing the increase of mitochondrial ROS by inhibiting the increased Plk3 protein in the diabetes-induced environment.

## 2 Materials and methods

### 2.1 Cells and cell lines, reagents, and gene silencing

All cell lines were sourced from commercial vendors. Beta TC-6 and Beta TC-tet mouse islet cell lines were cultured in Dulbecco's modified Eagle's media (DMEM) with 15% FBS and Streptomycin and Penicillin. For cell culture, powdered media components were dissolved by various commercially available water solutions (Evian, Smartwater, Aquafina, Fiji, Icelandic, Evamor, Eternal, Dasani, Crystal Geyser, Dr. coa (COA water), Poland Spring, Nestle pure life, Essentia, Ice mountain). All commercial waters used in the study were purchased online (internet) or from the local grocery store (Rochester, MN). And then, its medium was sterilized immediately by filtration using a membrane with a porosity of 0.2 μm. DMEM medium powder (Millipore-Sigma, Burlington, Mass.) was well diluted at room temperature in 14 different commercial sterilized drinks of water. The inside of the pack was thoroughly rinsed with sterile water to ensure that all the medium powder was used. The medium filled with water to a total volume of 1L was filtered and immediately sterilized using a 0.2-μm membrane bottle-top filters filter (Millipore-Sigma, Burlington, MA). 2′,7′-Dichlorofluorescein diacetate (DCF-DA), Propidium Iodide (PI, P4864), N-acetylcysteine (NAC), H_2_O_2_, stigmatellin, malonate, rotenone, and sodium azide were purchased from Sigma-Aldrich. MitoSOX, and 5,5′,6,6′-tetrachloro-1,1′,3,3′-tetraethylbenzimidazolylcarbocyanine iodide (JC-1) were obtained from Molecular Probes (Eugene, OR). Paraformaldehyde Solution (MFCD00133991) and 4′,6-diamidino-2-phenylindole (DAPI, 62248) were purchased from Thermo Scientific. MG132 (Z-Leu-Leu-Leu-al, S2619) were obtained from Selleck Chemicals. *Plk3* lentiviral shRNAs and the negative *control* shRNA were purchased from Creative Biogene Company. Please see Supplemental material.

### 2.2 Measurement of cell growth curve and DAPI staining

Beta TC-6 or Beta TC-tet cells were cultured in a medium prepared with general water (Control) or natural COA water and treated with streptozotocin for 72 h. Cell viability was also measured using a 3-(4,5-dimethylthiazol-2-yl)-2,5-diphenyltetrazolium bromide (MTT) assay. Cells were harvested and stained with MTT solution for three days, and absorbance was measured using a Fluorescent Microplate Reader (GENios Plus, Tecan). Cells were fixed in phosphate-buffered saline (PBS, Gibco-Invitrogen) with 3.7% paraformaldehyde (Sigma-Aldrich) at room temperature for 10 min. After three washings by 1X PBS, cells were incubated with 0.5 mg/ml DAPI solution (to measure apoptotic cell death) in PBS buffer for 10 min. Then cells were washed with PBS twice and examined with a fluorescence microscope under UV light.

### 2.3 Western blot analysis and antibodies

Protein lysate samples from two different pancreatic beta-cell lines were lysed by RIPA lysis buffer on ice supplemented with protease inhibitors, including 1 mM PMSF. Protein lysate samples were separated by preparing stacking gel and 10–15% separating gel during 4–8 h, and then transferred onto PVDF membranes. The membranes were blocked in TBS-T buffer in 5% fat-free milk for 1 h at room temperature. Next, the PVDF membrane was incubated with primary antibodies. The membrane was washed three times for 1% TBST and subsequently incubated for 1 h with related rabbit anti-mouse IgG-HRP secondary antibodies (Abcam). Mouse monoclonal antibodies recognizing Cleaved Caspase-8 (Asp384, #9748) and Cleaved Caspase-9 (Asp353, #9509) were obtained from Cell Signaling. Rabbit polyclonal antibodies recognizing Cleaved PARP (Asp214, ab32064) and Plk2 (ab137539) were obtained from Abcam. Rabbit polyclonal antibody recognizing Cleaved Caspase-3 (Asp175, #9661) was purchased from Cell Signaling. Mouse monoclonal antibody recognizing Plk1 (35–206) and Rabbit polyclonal antibody recognizing Plk3 invitrogen (PA5-97143) were purchased from Invitrogen. Anti-beta-actin mouse antibodies were purchased from Sigma. Please see Supplemental material.

### 2.4 Flow cytometric analysis

For analysis of cell cycle profile by FACS, cells were harvested in a time-dependent manner after induction. Pancreatic Beta TC-6 islet cells were cultured in a medium prepared with general water (Control) or natural COA water and then treated with streptozotocin for 72 h. And then, cells were fixed with ethanol, stained with propidium iodide (PI, 50 μg/ml, Sigma-Aldrich) for Sub-G1 as apoptosis (or CM-H2DCFDA (5 μg/ml) for ROS) containing RNase A (100 mg/ml, Sigma-Aldrich) for 30 min at room temperature. The DNA content was analyzed using a FACScan flow cytometer (Becton Dickinson, CA). The experiment was conducted three times independently.

### 2.5 Measurement of ROS production and immunofluorescence assay

Beta TC-6 were cultured in a medium prepared with general water (Control) or natural COA water and treated with Streptozotocin (STZ) for 18 h. The cells were stained with CM-H2DCFDA (5 μg/ml) or MitoSOX (5 μM) for 30 min and then observed by fluorescence microscopy. For immunofluorescence assays, cells were fixed in 3.7% paraformaldehyde (Sigma-Aldrich) for 10 min at room temperature. For immunofluorescence assays, Beta TC-6 cells were transfected with the indicated plasmids (Flag or Flag-Plk3) or were infected with the indicated plasmids (Control shRNA or Plk3 shRNA). And then cells were cultured in a medium prepared with general water (Control) or natural COA water and then treated with streptozotocin for 18 h. Cells were fixed in 3.7% paraformaldehyde for 10 min and permeabilized with 0.2% Triton X-100 for 10 min. The cells were then incubated for 24 h at 37 °C with rabbit polyclonal *anti*-Plk3 (Invitrogen) using standard protocols. 100–200 cells were monitored in each experiment by fluorescence microscopy and validated as described previously to quantify ROS or protein translocation. The experiment was conducted three times independently. Intracellular ROS production was measured using a fluorescence microscope (Olympus LX71 microscope), and the images were analyzed using MetaMorph software (Universal Imaging, Westchester, PA). To determine which mitochondrial electron transport chain processes are regulated by STZ treatment, we cultured cells in the presence of an antioxidant (NAC), rotenone (10 μM), Malonate (100 μM), Stigmatelin (1 μM), Sodium azide (1 mM), for 4 h. And cells were stained with MitoSOX (5 μM) for 30 min and harvested for immunofluorescence analysis. Green (or red) fluorescence was quantified using an excitation wavelength of 480 nm (red, 572 nm) and an emitter bandpass of 535 nm (red, 600 nm). Fluorescent images from multiple fields of view were captured, and ROS fluorescence intensity was normalized based on the cell number.

### 2.6 Measurement of mitochondrial membrane potential (ΔΨm)

To determine changes in ΔΨm, Islet Beta TC-6 cells were cultured in a medium prepared with general water (Control) or natural COA water or treated with streptozotocin for 18 h. And then cells were incubated with JC-1 (5 mM) for 30 min at 37 °C and washed twice with JC-1 staining buffer. JC-1 was excited at 490 nm. JC-1-stained cells were examined, using a fluorescence microscope (Olympus LX71 microscope), for excitation at 510–560 nm and with an emission filter for 570–620 nm. Emission fluorescence was filtered, and images were collected for FITC (green, 530 nm) channels using the FACScan flow cytometer (Becton Dickson). Fluorescence was analyzed with MetaMorph software (Universal Imaging, Westchester, PA, USA). Mitochondrial depolarization was analyzed as a decrease in the red/green fluorescence intensity ratio. The experiment was conducted three times independently.

### 2.7 Transient transfection, viral infection, and stable transduction

For transient overexpression studies, Beta TC-6 cells are first discarded of the existing serum-containing medium, rinsed 3 times with 1xPBS, and then replaced with a serum-free medium and left for 5 min. Then, the cells were transiently transfected with Flag or Flag-tagged Plk3 with a transfection reagent such as lipofectamine 2000 (Invitrogen). After 6 h, discard the serum-free medium containing the transfection reagent and rinse again with 1XPBS. After changing the medium to which fresh serum has been added, the cells are cultured for 24 h, and then, gene expression is checked under a fluorescence microscope. For viral infection, Beta TC-6 cells were performed at a cell density of 60–70%, and the cell culture medium was pretreated with polybrene one day before. *Plk3* lentiviral shRNAs (Creative Biogene) were infected with cells for 12 h, and then replaced with fresh medium. Cells infected with 2 μg/ml puromycin (Sigma-Aldrich) were selectively harvested. *Control* shRNA was simultaneously performed as a negative control.

### 2.8 3D organoid assay and insulin measurement

For 3D organoid assays, mouse islet Beta TC-6 or Beta TC-tet cells were cultured on NanoCulture plates (Scivax, Japan). After seeding cells, cells were cultured in the same condition with a 2D culture as cells were cultured in a medium prepared with 14 general or natural COA waters which were purchased internet online or from the local grocery store and treated with streptozotocin for 72 h. Cell sphere numbers were observed and recovered 0, 3, 5, and 7 days. Cells were incubated with 3 mM glucose or 25 mM glucose in KRBH buffer for 1 h. Glucose-stimulated insulin secretion of the supernatant medium was measured by Crystal Chem ELISA kit (Downers Grove, IL) and 3D organoid assay was described previously [31,32].

### 2.9 Statistical analysis

At least three independent replicates were assessed for each of the *in vitro* or *ex vivo* experiments, and pooled data were presented as mean ± standard error. Results were expressed as individual data points or as the mean ± S.D. Statistical analyses were performed using GraphPad Prism software (version 6.0; GraphPad Software, San Diego, CA). Statistical comparisons of scatter plots and bar graphs were performed by using either the one-way Analysis of Variance (ANOVA) or 2-way ANOVA for multiple comparisons with Holm-Sidak post hoc test. A P-value of *<0.05* was considered significant. Statistical significance was defined as P < 0.05(*), P < 0.01(**), and P < 0.001(***), ns: not significant.

## 3 Results

### 3.1 Natural COA water protects β cell survival in streptozotocin (STZ)-induced diabetes

**Fig. 1:**
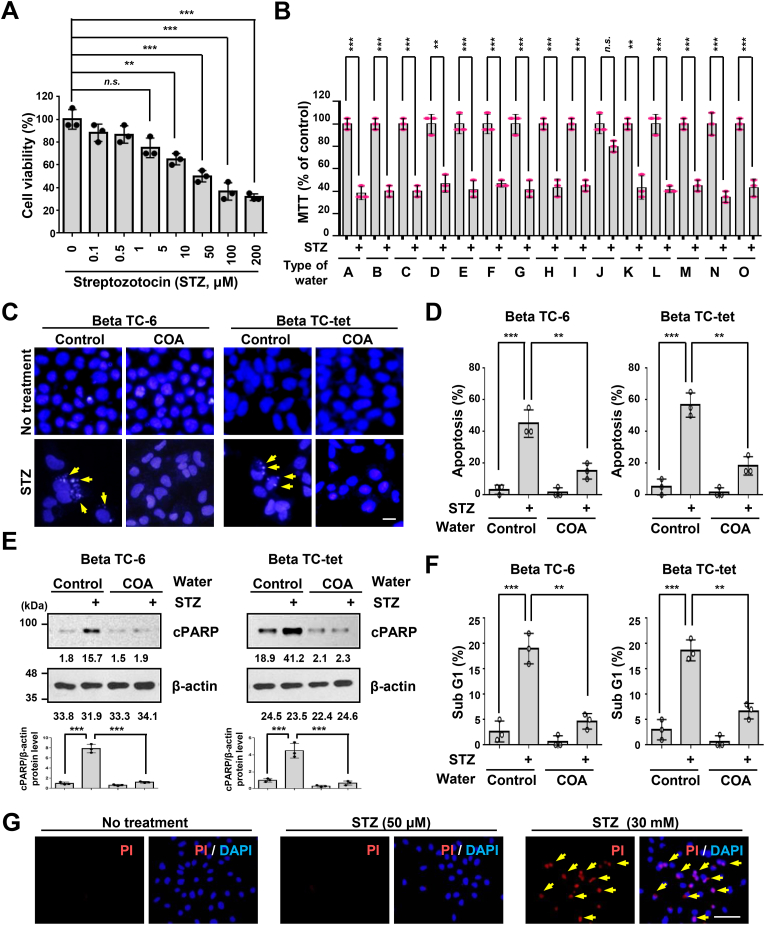
Natural COA water protects two mouse β cell survival in streptozotocin (STZ)-induced diabetes. (A, B) Beta TC-6 or Beta TC-tet cells were cultured in a medium prepared with indicated waters and treated by Streptozotocin (STZ) for 72 h. Cells were either directly counted (A) or counted by MTT assay (B). Each capital letter indicates differential commercial water brands in B. *A, Evian; B, Smartwater; C, Aquafina; D, Fiji; E, Icelandic; F, Evamor; G, Eternal; H, Dasani; I, Crystal geyser; J, Dr. coa (natural COA); K, Poland spring; L, Nestle pure life; M, Essentia; N, Ice mountain; O, commercial media (DMEM, Gibco)*. The results represent the means (±SD) of three independent experiments performed in triplicate. *P < 0.05, **P < 0.01, ***P < 0.001. Statistical comparison of scatter plot and bar graph was performed by repeated measure ANOVA with multiple comparisons test; *n.s.*, non-specific. (C, D) Pancreatic Beta TC-6 or Beta TC-tet cells were studied in the same manner as in B. Cells were cultured in a medium prepared with general water (Control) or natural COA water and then treated with streptozotocin for 72 h. The cells were stained with 4′,6-Diamidino-2-phenylindole dihydrochloride (DAPI) for apoptosis and then observed by fluorescence microscopy. Yellow arrows indicate programmed cell death (PCD), apoptotic bodies in (C). The representative images for each stain and quantification of DAPI-positive cells are shown in (C) and (D). **P < 0.01, ***P < 0.001. Statistical comparison of scatter plot and bar graph was performed by repeated measure ANOVA with multiple comparisons test. (E) Beta TC-6 or Beta TC-tet cells were cultured in a medium prepared with general water (Control) or natural COA water. Some of these cell groups were treated by STZ for 72 h. Cell lysates were subjected to immunoblot as indicated. cPARP, cleavage PARP. Relative protein band intensities in Western blot were quantified by using ImageJ software and the cPARP/β-actin ratio was determined. And then the statistical comparison of scatter plot and bar graph was performed by repeated measure ANOVA with multiple comparisons test. ***P < 0.001. (F) Beta TC-6 or Beta TC-tet cells were cultured in a medium prepared with indicated waters and half of those cell group were treated by STZ for 72 h. Cells were analyzed for sub G1(apoptosis) by flow cytometry (FACS). **P < 0.01, ***P < 0.001. Statistical comparison of scatter plot and bar graph was performed by repeated measure ANOVA with multiple comparisons test. (G) Beta TC-6 cells were treated with either 50 μM or 30 mM STZ for 30 min. Beta cells in culture were stained with Propidium Iodide (PI, 1 μg/ml) for 10 min and then were fixed with 4% paraformaldehyde. Then, the cells were again stained with 4′,6-diamidino-2-phenylindole (DAPI, 1 μg/ml) and observed under a microscope. Representative images of necrosis (PI) in the Beta TC-6 cells. Scale bars, 20 μm. All experiments were repeated independently at least three times with similar results. (For interpretation of the references to colour in this figure legend, the reader is referred to the Web version of this article.)

Previous studies reported that STZ stimulates pancreatic β-cell apoptosis *in vitro* [1,6,7]. To confirm the viability of pancreatic β-cell for the diabetes stress model, we tested two different Beta TC-6 or Beta TC-tet islet cells after STZ treatment. We found that the IC_50_ of Beta TC-6 was 50 μM using MTT assay (Fig. 1A). Similar to the results in other pancreatic cells, Beta TC-tet (data not shown) demonstrated that STZ strongly affected pancreatic β-cell death (Fig. 1A). Next, we hypothesized that some specific water might be able to prevent diabetes progression. To satisfy this hypothesis, we cultured beta cells by preparing a cell culture solution treated with STZ in a medium prepared with 15 different drinks of water currently being sold. Through this experiment, we found that most of the general waters had the same effect on beta-cell death by STZ induction as the water in the current culture medium (Fig. 1B). On the contrary, it was confirmed that the STZ-induced apoptosis effect on these beta cells was protected only in a medium prepared with natural COA water (Fig. 1B and Supplemental data). We further found that general water (Control) does not protect apoptosis to beta TC-6 cells induced by STZ, whereas natural COA water inhibited it (Fig. 1C and D). The same results were obtained in other islet cells (Fig. 1D). Immunoblot analysis of beta-cell lysates from general water and natural COA water revealed that activated-PARP1 protein levels were decreased in cells incubating natural COA water (Fig. 1E). We also used flow cytometry, which is a method of predicting the cell cycle and cell state by fractionated DNA content, where a value of sub-G1 means apoptosis. In our study, the sub-G1 population of beta cells increased by STZ treatment was reduced by natural COA water (Fig. 1F). Furthermore, we tested whether STZ cell death was also affected by necrosis. Beta cells treated with STZ with 30 mM showed necrosis, whereas necrosis was not observed with STZ at 50 μM, which we found as IC50 (Fig. 1G). This means that necrosis had no effect on cell death caused by 50 μM of STZ. These results suggest that natural COA water may have activity against STZ-induced diabetes and cell death.

In a previous study, we analyzed the permeability of water using frog eggs to determine the nature of COA natural water [33,34]. As a result, natural COA water increased the permeability of aquaporin (AQP) 1 and 7 (water channel proteins in the cell membrane) by 1.6 and 1.5 times, respectively, which are different from other waters (Data not shown). However, the permeability of AQP 2, 3, 4, and 5 was relatively similar to the control. Aquaporins are channel proteins located in cell membranes that help transport water and are widely distributed in several gene types (AQP1-12) in the human body and play an important role in the pathogenesis of many diseases in the absence of genes [[24], [25], [26],^28^,^35^]. Especially, AQP1 gene is highly expressed in renal tubules and microvessels, choroid plexus, ciliary epithelium, corneal endothelium, pain-processing C-fibers, and vascular endothelium, tumor vessels and red blood cells. The absence of this gene may cause diuresis, reduced tumor angiogenesis, reduced intraocular pressure, reduced CSF secretion, and reduced nociception in humans. AQP7 is widely distributed in fat cells, renal proximal tubule (S3 segment), testis, and myocardium, and when this gene is absent, causes obesity, insulin resistance, and hyperglyceroluria. Since several papers have already dealt with the correlation between aquaporin and diabetes [25,30,35,36], we tried to find the cause in other fields such as programmed cell death, cell proliferation, cell cycle, ROS, mitochondrial function, and cell metabolism.

### 3.2 Loss of mitochondrial membrane potential (ΔΨm) through ROS is regulated by natural COA water under diabetic stress

**Fig. 2:**
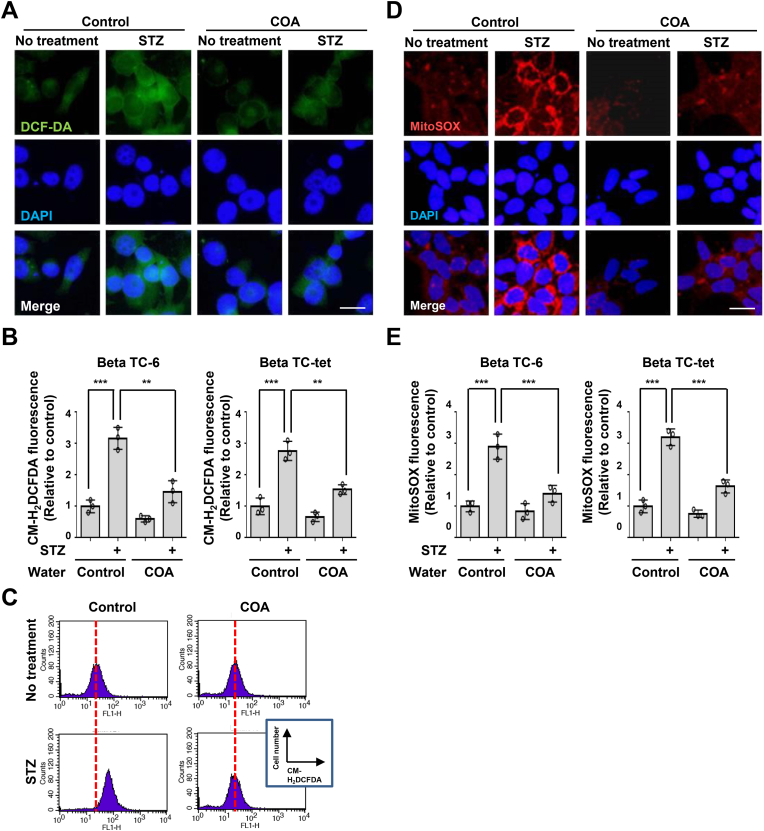
STZ-induced ROS generation in beta cells is blocked by natural COA water. (A, B) Beta TC-6 or Beta TC-tet pancreatic cells were cultured in a medium prepared with general water (Control) or natural COA water and then half of those cell group were treated by STZ for 18 h. The cells were stained with CM-H_2_DCFDA (5 μg/ml) for 30 min and then observed by fluorescence microscopy. For quantification, the computer overlaid the images obtained from fluorescence microscopy, and CM-H_2_DCFDA fluorescence was analyzed with Meta Morph software. Representative images of ROS in the Beta TC-6 cells. Scale bars, 20 μm (A). The results represent the means (±SD) of three independent experiments performed in triplicate. Statistical comparison of scatter plot and bar graph was performed by repeated measure ANOVA with multiple comparisons test; **P < 0.01; ***P < 0.001 (B). (C) Beta TC-6 islet cells were cultured in a medium prepared with general water (Control) or natural COA water. Other cells were also incubated in a medium prepared with indicated waters and then treated with streptozotocin for 18 h. The cells were stained with CM-H_2_DCFDA (5 μg/ml) for 30 min and then analyzed increased ROS generation by flow cytometry. All experiments were repeated independently at least three times with similar results. Red-dot lines indicate the peak of ROS curves in the non-STZ-treated cells. (D, E) Beta TC-6 or Beta TC-tet cells were cultured in a medium prepared with general water (Control) or natural COA water. Other cells were also incubated in a medium prepared with indicated waters and then treated with streptozotocin for 18 h. The cells were stained with MitoSOX (5 μM) for 30 min and then observed by fluorescence microscopy. Representative images of mitochondrial ROS in the Beta TC-6 cells (D). The measurement of mitochondrial ROS production (E). Statistical comparison of scatter plot and bar graph was performed by repeated measure ANOVA with multiple comparisons test; ***P < 0.001; *n.s.*, non-specific. All experiments were repeated independently at least three times with similar results.

**Fig. 3:**
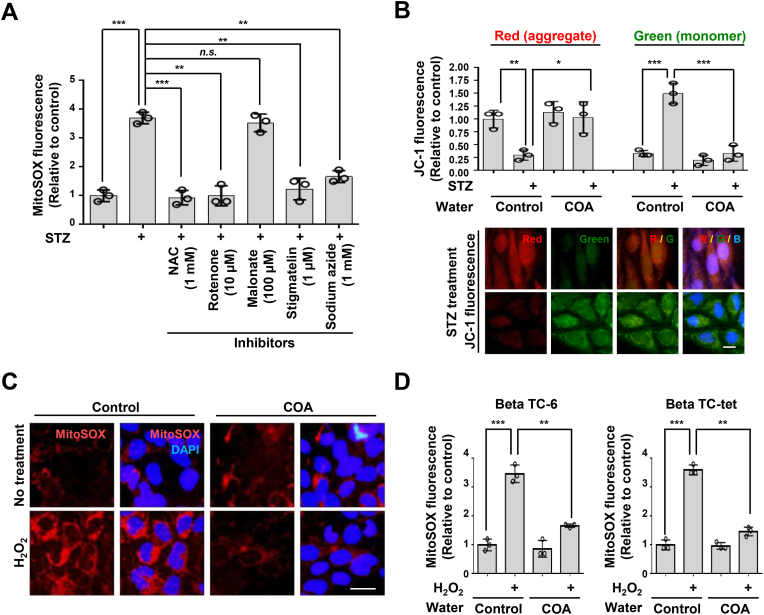
Natural COA water regulates mitochondrial membrane potential (*ΔΨm*) through reactive oxygen species. (A) Beta TC-6 cells were cultured in a medium prepared with general water and then treated by Streptozotocin for 18 h. Cells were cultured in the presence of NAC (1 mM), rotenone (10 μM), Malonate (100 μM), Stigmatelin (1 μM), Sodium azide (1 mM) for 4 h, and harvested for immunofluorescence analysis. The cells were stained with MitoSOX (5 μM) for 30 min and then observed by fluorescence microscopy. Representative images of mitochondrial ROS in the cells. 100 cells were monitored in each experiment. (B) Beta TC-6 cells were cultured in a medium prepared with general water (Control) or natural COA water and then treated by Streptozotocin for 18 h. Cells were incubated with the fluorescent probe JC-1 (5 mM) for 30 min and were observed red (JC-1 aggregates, polymer) and green (JC-1 monomers) fluorescence intensities by a fluorescence microscope. In healthy mitochondria, JC-1 forms aggregate with red fluorescence as a polymer. On the other hand, in the case of mitochondria with lower mitochondrial membrane potential, the form of JC-1 is converted to a monomer and is displayed as a green fluorescence signal. Mitochondrial depolarization was analyzed as a decrease in the red/green fluorescence intensity ratio. Scale bar, 20 μm. Data in (A) and (B) are presented as the mean ± SD of *n = 3* independent experiments. *P < 0.05, **P < 0.01, ***P < 0.001. Statistical comparison of scatter plot and bar graph was performed by repeated measure ANOVA with multiple comparisons test; *n.s.*, non-specific. (C, D) Beta TC-6 or Beta TC-tet pancreatic cells were cultured in a medium prepared with indicated waters and treated by Hydro peroxide (H_2_O_2_, 100 μM) for 30 min. The cells were stained with MitoSOX (5 μM) for 30 min and then observed by fluorescence microscopy. Representative images of mitochondrial ROS in the Beta TC-6 cells. Scale bars, 20 μm (C). The measurement of mitochondrial ROS production. The results represent the means (±SD) of three independent experiments performed in triplicate. Statistical comparison of scatter plot and bar graph was performed by repeated measure ANOVA with multiple comparisons test; **P < 0.01; ***P < 0.001 (D). (For interpretation of the references to colour in this figure legend, the reader is referred to the Web version of this article.)

Several papers suggested that diabetes results in increased ROS and oxidative stress, leading to pancreatic cell death in cells by multiple mechanisms and finally tissue damage [1,3,9,10,[13], [14], [15], [16], [17],^22^,^37^]. We examined STZ-induced ROS production change by staining beta cells with the oxidant-sensitive dye, 5-(and-6)-chloromethyl-2′,7′-dichlorodihydrofluorescein diacetate acetyl ester (CM-H_2_DCFDA), after natural COA water (Fig. 2A and B). STZ-induced ROS production was increased in general water-cultured control pancreatic cells while with natural COA water it was significantly blocked. We obtained the same result from Beta TC-6 as for Beta TC-tet (Fig. 2B). We verified that natural COA water has antioxidant activity under the diabetes stage through flow cytometry (Fig. 2C), suggesting that downregulation of ROS generation by natural COA water may play an essential role in controlling diabetes. To confirm whether increasing ROS by STZ in beta cells came from mitochondria, we used the MitoSOX probes which are widely used to detect mitochondrial ROS as superoxide (Fig. 2D and E). Mitochondrial ROS was detected in STZ-treated cells, and the numerical value was about three times higher than that of the control group. Interestingly, STZ-induced mitochondrial ROS elevation did not increase in beta cells incubated on natural COA water. These results were similar for other Beta cells (Fig. 2E). The mammalian mitochondrial electron transport chain (ETC) includes complexes I-IV and is the site of oxidative phosphorylation in eukaryotes [15,16]. There are two electron transport pathways in ETC such as Complex I/III/IV with NADH as a substrate and Complex II/III/IV with succinate as a substrate. The ETC utilizes a series of electron transfer reactions to generate cellular ATP through oxidative phosphorylation. The increased ROS generated here interferes with the regulation of cell homeostasis and worsens the mitochondrial state, increasing intracellular stress. To determine which mitochondrial electron transport chain processes are regulated by STZ, we cultured cells in the presence of an antioxidant (NAC), mitochondrial respiratory chain complex I inhibitor (rotenone), complex II inhibitor (malonate), complex III inhibitors (stigmatelin), or complex IV inhibitor (sodium azide), and mitochondrial ROS was assessed by immunofluorescence assay (Fig. 3A). We found that STZ-induced ROS was blocked by rotenone, stigmatelin, sodium azide, and NAC. In contrast, other inhibitor, Malonate failed to inhibit STZ-induced mitochondrial ROS generation. These data demonstrated that the generation of mitochondrial ROS by STZ treatment in beta cells is type I/III/IV of mitochondrial chains. Since the mitochondrial membrane potential (ΔΨm) plays a key role in ATP production, it is widely used as a crucial indicator to check the health status of cells. In other words, a decrease in MMP causes a change in pH in the mitochondrial intermembrane space and matrix, which in turn means that ATP production, is inhibited. The JC-1 fluorescent dye is analyzed by dividing it into green or red fluorescence yield depending on the MMP. It is a suitable method for distinguishing cells with high or low mitochondrial potential. In Fig. 3B, we confirmed that STZ instigates a decrease in MMP by stimulating the mitochondrial membrane potential (ΔΨm). The mitochondrial dysfunction by STZ treatment increased 3–4 fold, meaning that STZ induces potent MMP dependent cytotoxicity in beta cells. But, the medium prepared with natural COA water prevents these effects, suggesting that natural COA water restores the loss of the mitochondrial membrane potential (ΔΨm) produced by STZ treatment. We conducted the reproducibility of the study on the antioxidant activity of natural COA water under the H_2_O_2_ induction experiment and obtained the same results as in the previous experiment (Fig. 3C and D).

### 3.3 Natural COA water negatively controls Plk3 expression under streptozotocin (STZ)-induced diabetes

**Fig. 4:**
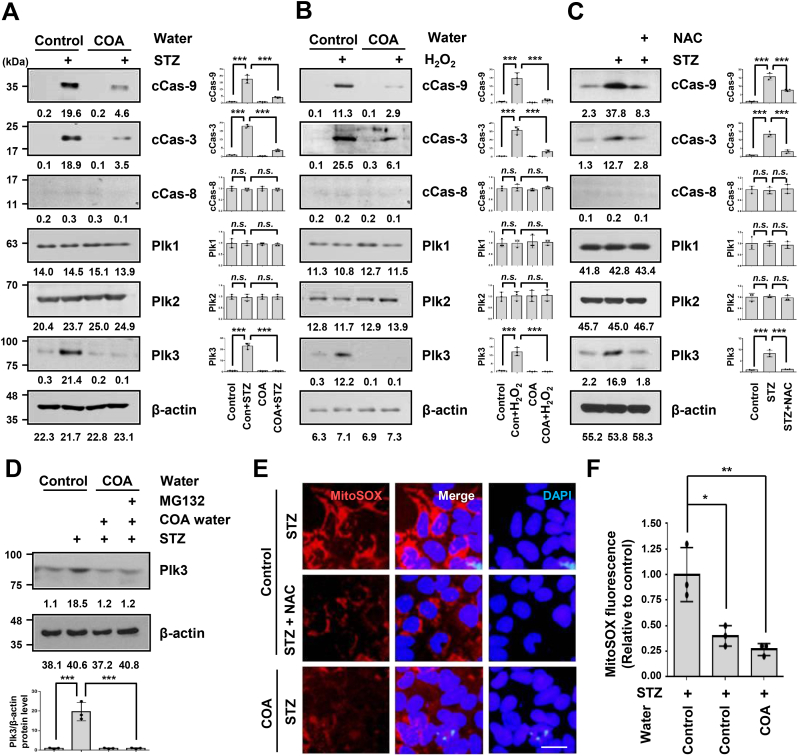
The mitochondria-mediated apoptotic cell death via streptozotocin (STZ) in beta cells is inhibited by natural COA water. (A) Beta TC-6 islet cells were cultured in a medium prepared with general water (Control) or natural COA water and then treated with streptozotocin for 72 h. Cell lysates were analyzed by immunoblotting with the antibodies indicated. (B) Beta TC-6 cells were cultured in a medium prepared with general water (Control) or natural COA water and then treated by H_2_O_2_ (10 μM) for 24 h. Cell lysates were analyzed by immunoblotting with the antibodies indicated. (C) Pancreatic Beta TC-6 islet cells were cultured with indicated waters and then treated by Streptozotocin or NAC (1 mM). Cell lysates were analyzed by immunoblotting with the antibodies indicated. (D) Beta TC-6 cells were cultured in a medium prepared with general water or natural COA water and then treated with streptozotocin for 72 h. And some of these cells were treated with MG132 (10 μM, for 4 h) in a medium prepared with natural COA water. Cell lysates were analyzed by immunoblotting with the antibodies indicated. (E, F) Beta TC-6 cells were cultured in a medium prepared with general water (Control) or natural COA water and then treated with streptozotocin for 18 h. The cells were stained with MitoSOX (5 μM) for 30 min and then observed by fluorescence microscopy. Representative images of mitochondrial ROS in the Beta TC-6 cells (E). Quantification of cleaved Caspase-9, cleaved Caspase-3, cleaved Caspase-8, Plk1, Plk2, and Plk3 levels relative to β-actin levels. The signal of the band was quantified with Image J software. cCas-9, Cleaved Caspase-9; cCas-3, Cleaved Caspase-3; cCas-8, Cleaved Caspase-8. Statistical comparison of scatter plot and bar graph was performed by repeated measure ANOVA with multiple comparisons test; *P < 0.05, **P < 0.01, n.s., non-specific. All experiments were repeated independently at least three times with similar results.

**Fig. 5:**
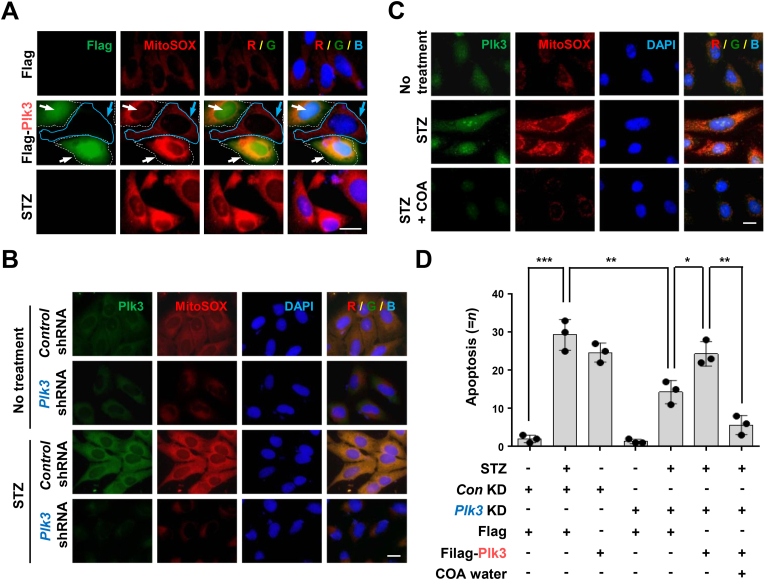
Plk3 induces apoptosis through mitochondrial ROS under streptozotocin (STZ)-induced diabetes stress, but natural COA water negatively controls Plk3 expression. (A) Beta TC-6 cells were transfected with the indicated plasmids (Flag or Flag-Plk3) and then cells were stained with MitoSOX (5 μM) for 30 min. Cells were analyzed by immunofluorescence assay with the Flag antibody and then observed by fluorescence microscopy. Representative images, scale bar, 20 μm. All experiments were repeated independently at least three times with similar results. (B) Beta TC-6 cells were infected with the indicated plasmids (*Control* shRNA or *Plk3* shRNA) and then treated with streptozotocin for 72 h and cells were stained with MitoSOX (5 μM) for 30 min. For immunofluorescence assays, cells were fixed, permeabilized, incubated with *anti*-Plk3 for 24 h and then cells were observed fluorescence microscopy. Scale bar, 20 μm. All experiments were repeated independently at least three times with similar results. (C) Beta TC-6 cells were cultured in a medium prepared with general water (Control) or natural COA water and then treated with streptozotocin for 18 h. The cells were stained with MitoSOX (5 μM) for 30 min and incubated with *anti*-Plk3 and then observed by fluorescence microscopy. Representative images, scale bar, 20 μm. (D) Beta TC-6 cells were infected with the indicated plasmids (*Control* shRNA or *Plk3* shRNA) and then treated with streptozotocin for 72 h. And some of these cells were transfected again with the indicated plasmids (Flag or Flag-Plk3) in a medium prepared with general water (Control) or natural COA water. The measurement of apoptotic cell numbers. Data is presented as the mean ± SD of *n = 3* independent experiments. Statistical comparison of scatter plot and bar graph was performed by repeated measure ANOVA with multiple comparisons test; *P < 0.05, **P < 0.01, ***P < 0.001.

Mitochondria also play an essential role in activating apoptosis in mammalian cells [38]. During apoptosis in cells, cytochrome *c* is released from the mitochondria to the cytosol through the action of the proteins Bcl-2, Bcl-XL, Bax, Bid, and Bak [14,16,38]. Mitochondrial apoptosis is an evolutionarily extremely well-conserved suicide mechanism that binds cytochrome *c* and caspase-9 and activates caspase-3 ^16, 38^. A previous study reported that STZ treatment enhanced apoptosis by significantly increasing intracellular ROS, Bax, and activated-caspase-9 and -3 levels in pancreatic MIN6 cells [12]. We tried to investigate the specific mechanisms through immunoblotting (Fig. 4). There was an increased cleaved caspase-9 and -3 protein levels in the STZ-treated control group cells but not in a medium prepared with the natural COA water cultured cells (Fig. 4A). We further confirmed that there is no caspase-8-related cell death mechanism in this experiment. Several recent papers have reported that the Plk family genes are closely related to diabetes and ROS, and have signaling pathways involved in autophagy, apoptosis, and inflammation [[20], [21], [22], [23],^39^]. Indeed, we observed significantly increased Plk3 protein expression, unlike Plk1 and Plk2, in STZ-treated pancreatic cells (Fig. 4A, B, and C). Yet, the high protein expression of the Plk3 was not observed in a medium prepared with the natural COA water, demonstrating that COA water negatively controlled the protein stabilization of Plk3. To investigate whether the pancreatic intracellular apoptosis mechanism is due to increased intracellular ROS, we treated the cells with H_2_O_2_. Similarly, natural COA water was also cultured under the same conditions to confirm the change (Fig. 4B). In the pancreatic cells treated with H_2_O_2_, the levels of cleaved proteins, which are proteins activated by caspase-9 and -3, were significantly increased, as in the STZ-treated cells shown in Fig. 4A. Changes in these proteins were inhibited by natural COA water. Furthermore, to confirm that STZ-induced apoptosis increased intracellular ROS, we demonstrated that the function was inhibited using NAC, an antioxidant inhibitor, after STZ treatment (Fig. 4C). We confirmed that natural COA water not only reduced the endogenous Plk3 protein, but also blocked the Plk3 protein increased by external stress such as STZ or H_2_O_2_ in Beta TC-6 cells (Fig. 4A, B, and C). To determine whether natural COA water can degrade Plk3 protein, we treated Beta TC-6 cells with MG132, a proteasome inhibitor under the same conditions as in Fig. 4 (Fig. 4D). STZ markedly increased the level of Plk3 protein in beta cells, and the increase in this protein was reduced by natural COA water. However, the effect of reducing the Plk3 protein by the natural COA water was not changed at all despite the MG132 treatment. These data suggest that the negative regulatory function of natural COA water for increased Plk3 under STZ stress is transcriptional or related to signal transduction rather than protein level as translational. Also, we conducted the reproducibility of the study on the antioxidant activity in a medium prepared with natural COA water under STZ treatment and obtained the same results as the NAC treatment (Fig. 4E and F). Since the protein of Plk3 was significantly increased by STZ in this experiment in Fig. 4A, B and C, we hypothesized that expression of Plk3 would regulate mitochondrial ROS and apoptosis (Fig. 5). We first found that Plk3 overexpressed cells significantly increased mitochondrial ROS compared to non-transfected normal beta cells (Fig. 5A). The expression level of Plk3 protein in normal beta cells under fluorescence microscopy was very low, and mitochondrial ROS was also not very bright but both signals were increased in the STZ-treated pancreatic cells (Fig. 5B), consistent with the protein levels pattern on a Western blot of the previous Fig. 4 results. In the absence of Plk3, mitochondrial ROS production was dramatically reduced, and this result was more pronounced in STZ-treated cells. Intriguingly, expression of Plk3 protein and mitochondrial ROS increased by STZ were significantly decreased in a medium prepared with natural COA water (Fig. 5C). Furthermore, we confirmed whether Plk3-generated mitochondrial ROS leads to apoptosis in STZ-treated beta cells and investigated whether Plk3 gene deletion and natural COA water could inhibit its effect. A significant number of apoptotic cells were observed in the STZ-treated cells and in the cells overexpressed with Plk3 (Fig. 5D). We also found that apoptosis in the beta cells treated with STZ was significantly reduced by *Plk3* knockdown. To more prove that *Plk3* shRNA prevents STZ-induce apoptosis, we expressed with stably expressing Plk3 construct (or empty vector) under STZ-treated cells and counted apoptotic cells. Apoptosis by mitochondria ROS was decreased in STZ-treated cells stably expressing *Plk3* shRNA, while reconstitution with shRNA-resistant Plk3 restored apoptotic cell death (Fig. 5D). Interestingly, however, this re-increase of apoptosis by this reconstitution with *Plk3* shRNA-resistant Plk3 induction was re-inhibited by natural COA water. These data suggest that Plk3-mediated apoptosis is closely related to the activation of mitochondria ROS and that natural COA water inhibits it (Fig. 5A, B, and D).

### 3.4 Natural COA water positively affects the regulation of insulin secretion under STZ-induced diabetes in the 3D organoid model

**Fig. 6:**
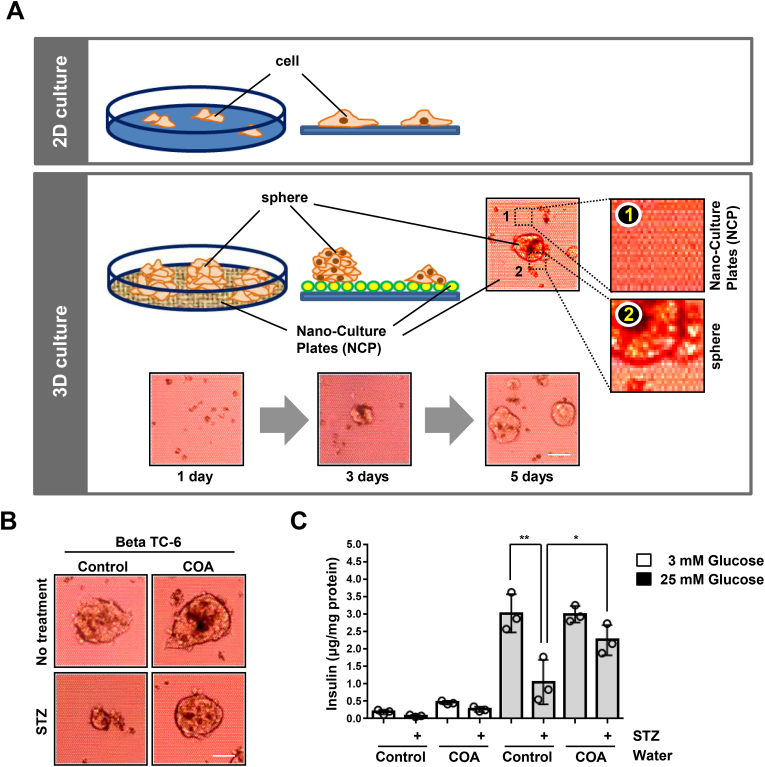
The regulation of insulin secretion under STZ-induced diabetes stress in the 3D organoid model is positively affected by natural COA water. (A) The schematic model for experimental cell culture design. Unlike 2D (top), 3D organoid cell culture (bottom) contains special nanomaterial at the bottom of the culture dish, so the cells grow more stably and are almost formed in the shape of real cells like the human organ or cells. Two islet beta (Beta TC-6, Beta TC-tet) cells are made from the sphere formation in 3D cell culture because of insulinoma. Representative images, scale bar, 20 μm. (B) Beta TC-6 pancreatic cells were cultured in a medium prepared with general water (Control) or natural COA water and then half of those cell group were treated by STZ for 72 h. The representative images for each cell are shown in B. Representative images, scale bar, 20 μm. All experiments were repeated independently at least three times with similar results. (C) Beta TC-6 islet cells were cultured in a medium prepared with general water (Control) or natural COA water and then treated with streptozotocin for 72 h. And then cells were incubated with 3 mM glucose or 25 mM glucose in KRBH buffer for 1 h. Glucose-stimulated insulin secretion of the supernatant medium in Control or natural COA water-cultured islets (*n = 3* per group) was measured by ELISA kit. Data is presented as the mean ± SD of *n = 3* independent experiments. *P < 0.05, **P < 0.01. Statistical comparison of scatter plot and bar graph was performed by repeated measure ANOVA with multiple comparisons test.

**Fig. 7:**
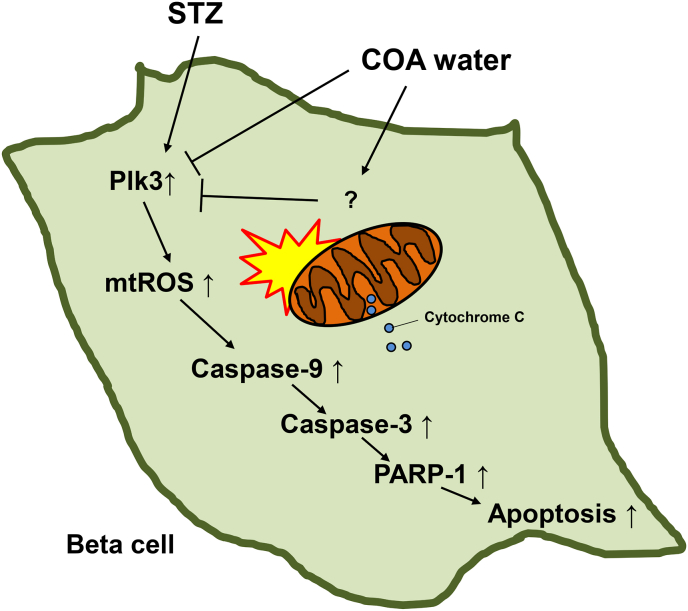
Natural COA water protects the pancreatic beta cells via inhibition of mitochondrial ROS under STZ-induced diabetes stress. The schematic model of mitochondrial-mediated apoptosis and natural COA water effect. Streptozotocin (STZ) for diabetes stress induces apoptosis in pancreatic beta cells. Plk3 is activated by STZ to regulate mitochondrial ROS generation. These events lead to low mitochondrial membrane potential (ΔΨm) and mitochondrial-mediated apoptosis including caspase-9, cytochrome *c*, ATP, caspase-3, and PARP-1. Natural COA water negatively controls Plk3 expression under streptozotocin (STZ)-induced diabetes stress and inhibits mitochondrial ROS and apoptotic cell death in pancreatic beta cells.

Next, we examined the insulin secretion of these pancreatic beta cells in *ex vivo* models after stimulation with glucose (Fig. 6). Since each tissue and various cells in the human body are composed of many complex shapes, we needed to establish the best biological environment like the human body for accurate data and analysis. 3D cell culture is one of the best culture systems that help cells to function by providing the optimal natural environment directly to the cells so that the cells can live in an environment similar to the natural human body [32,40]. This culture method is distinguished from the 2D method in that it helps the optimal growth of cells by using a scaffold such as collagen or other matrix or attaching a nanomaterial to the surface of a cell culture dish (Fig. 6A). We used 3D spheroid culture using nanoplates for this experiment, and three days after seeding the cell lines, all pancreatic cells began to form spheroids (Fig. 6A). We tried the 3D organoid tissue culture system to confirm our results from Fig. 1, Fig. 2D that the medium prepared with natural COA water blocks STZ-induced apoptosis via ROS (Fig. 6B). Furthermore, we found that STZ-treated beta cells in general water showed lower insulin secretion of the supernatant medium by glucose stimulation than the control cells, but natural COA water-cultured cells still produced insulin in 3D culture systems (Fig. 6C). Thus, these data suggest that natural COA water plays a role in preventing diabetes as well as stabilizing the growth of beta cells and promoting insulin secretion (Fig. 7).

## 4 Discussion

In this study, we found natural COA water prevents STZ diabetic stress through the stabilization of pancreatic beta cells. There are several causes of diabetes, including mTOR mediated metabolic problems, glucotoxicity, inflammation, mitochondrial dysfunction, increased oxidative stress or ER stress, caused by various programmed cell death such as apoptosis, autophagy, or necroptosis [[1], [2], [3], [4],[6], [7], [8], [9], [10], [11]]. To check ROS, a major factor of diabetes, we treated the cells with STZ to activate abnormally increased ROS, which eventually led to apoptosis. We found that natural COA water, by blocking the abnormal mitochondrial ROS through STZ-induced diabetic stimulation, optimized metabolic function and blocked apoptosis in two pancreatic beta-cells (Fig. 1, Fig. 2). Natural COA water blocked abnormal mitochondrial ROS caused by STZ, optimizing metabolic function as well as blocking apoptosis. To study the function of natural COA water in more detail, we checked to see which ROS damaged cells in STZ treatment. This study found that natural COA water negatively controlled the unstable mitochondrial ROS, which mediate the electron transport chains I, III, and IV (Fig. 3A). Also, we confirmed that natural COA water blocked unstable mitochondrial membrane potential caused by the STZ diabetic stress condition (Fig. 3B). Mitochondria ROS has a prominent role in cell death through the interaction between mitochondria-related apoptotic factors including, Bcl-2, Bcl-XL, Bax, Bak, Bim, Bid, etc. When a mitochondria-mediated apoptotic signal comes, Caspase-8 activates Bax, Bak, Bim, Bid, releasing cytochrome *c* to the cytosol from the intermediated space within the mitochondria [14,16,38]. The cytochrome *c* binds to Caspase-9 and ATP to form an apoptosome which activates Caspase-3 leading to cell death by PARP cleavage in nuclei [16,38]. As such, mitochondrial ROS functions as an initiator of complex and diverse apoptotic processes using the mitochondria [3,[9], [10], [11],[14], [15], [16], [17],^37^]. As mentioned earlier, the increased STZ-mediated mitochondrial ROS was sufficiently suppressed with natural COA water.

Interestingly, we also found that Plk3 has an essential role in the mitochondria ROS under STZ diabetic stress. Plk3 is a serine/threonine kinase that is known to be significantly involved in cell division as well as cancer formation and DNA damage [18,37,39]. Particularly, in the DNA damage response (DDR) signaling pathway, Plk3 apoptosis is induced by p53, which is phosphorylated by the ATM protein, a DNA damage regulator for DNA double-strand breaks [39]. Meanwhile, one group reported that Plk3 was associated with diabetes-related cataracts [23], suggesting that excessive use of sorbitol affects DNA damage in human corneal epithelial cells caused by osmotic stress and that Plk3 gene expression is increased under galactose-induced diabetic cataract conditions [23]. However, there was still no specific study on how Plk3 affects pancreatic beta-cells under diabetes-related stress and by what mechanism. This study found that Plk3 protein levels were increased under diabetes-induced stress as STZ or ROS (Fig. 4, Fig. 5B and D). This increased Plk3 acts as an essential signal for inducing apoptosis, affecting mitochondrial ROS as well as mitochondrial membrane potential (ΔΨm) stabilization from type I/III/IV of mitochondrial electron transport chain (ETC). Furthermore, we also found that most of the ROS caused by STZ diabetes-induced stress originate from mitochondria, and the increased ROS induce strong stress in cells and induces apoptosis. Similarly, the increased activity of proteins such as cytochrome *c* and activated caspase-9 were observed, and it was also found that the mechanism of apoptosis through mitochondria occurs (Figs. 4 and 5D). Another interesting finding is that Plk3 is one of the first proteins to respond to the STZ diabetic stress, and we can block the apoptosis pathway by regulating the amount of this increased protein. For reference, in this experiment, we could not detect changes in the Plk members Plk1 and Plk2 (Fig. 4A, B and C). We found through several specific experiments that natural COA water inhibited the initiating effector of Plk3 in the STZ diabetic stress environment, thereby pre-preventing unstable mitochondrial ROS and mitochondrial membrane potential (ΔΨm) destabilization, suggesting that natural COA water prevents apoptosis and ultimately protects pancreatic cells (Fig. Fig. 4, Fig. 5, Fig. 7).

It can be challenging measure insulin levels after culturing pancreatic cells *ex vivo*. In this study, our research team succeeded in culturing mouse pancreatic cells for more than 6 months through a special method called nano-culture plate (NCP), and at the same time successfully quantified insulin secreted into somatic cells in 3D organoid culture system (Fig. 6). Apoptosis occurred in pancreatic cells with the STZ environment on *ex vivo*, and the period occurred within 3–4 days after the STZ treatment. Although living cells were present, the amount of insulin was barely measurable. Here, we hypothesized that specific water types might play a crucial role in pancreatic beta-cell functions to prevent STZ diabetic stress. Among 14 different commercial drinks of water (general waters), we tested for the stability of pancreatic cells under STZ treatment. Among them, in only one medium prepared with natural COA water (Supplemental data, https://www.kigam.re.kr/english/), healthy pancreatic cells, as well as a certain amount of insulin, were measured in the setting of diabetic stress caused by STZ (Fig. 6B and C), suggesting that natural COA water may sufficiently protect against advanced STZ diabetic stimulation (Fig. 7). We had already commissioned a specialized Japanese institute (Kitagawa Scientific General Research Institute LLC.) [25,28], to identify the water characteristics of natural COA water in July 2017. The most representative method for distinguishing the properties of water is to check the type of aquaporin, a channel for water passage in the membrane of cells, using frog eggs [33,34]. After each injection of AQP1, 2, 3, 4, 5, or 7 into xenopus oocytes, natural COA water or general water was supplied to measure water permeability. We demonstrated that the permeability of AQP1 and 7 (water channel proteins in the cell membrane) was relatively increased, unlike general water (Data not shown). AQP1 and 7 are specifically involved in activating systemic organ functions, immunity, and lipid metabolism. This report suggests that long-term drinking of natural COA water could have health maintenance applications. In addition, this report suggests that studies on changes in blood moisture in the human body and cell activity of individual organs related to natural COA water are necessary. Here, our research team has clearly demonstrated that natural COA water improves insulin secretion from pancreatic beta cells even in a STZ diabetic stimulation (Fig. 6). Mechanically, STZ diabetic stress induces an abnormal increase in the Plk3 protein expression level, which promotes abnormal intracellular mitochondrial ROS involved in apoptosis. At this time, natural COA water maintains the stabilization of pancreatic cells by directly or indirectly inhibiting Plk3 that is increased in this STZ diabetes-related stress (Fig. 7). Another possibility is that STZ-induced apoptosis increases the expression of Plk3 as well as numerous DNA damage repair-related genes and proteins that affect DNA damage, but at this time natural COA water may block one of these mechanisms to inhibit STZ-induced apoptosis [18,37,39]. A similar case has already been reported in Hita tenryosui water in Japan [25,27,29]. The study found that the natural water removes intracellular ROS and inhibits tumor angiogenesis. It is also reported that this natural water can alleviate the symptoms of type I diabetes model mice induced by alloxan by inhibiting oxidative damage to pancreatic cells [25,27,29].

Our study did not check for various genes or proteins levels related to apoptosis, autophagy, necroptosis, pyroptosis and other metabolic-related genes. Also, how natural COA water regulates Plk3 protein levels and which E3 ligases or deubiquitinases are involved remains unresolved. In addition, further research on how natural COA water differs from other general water is necessary. We have not yet found any differences in composition between natural COA water and other general waters. We speculate that natural COA water will have special water formation and structural changes compared to other general waters, and further research is ongoing [28]. Also, we hypothesize that the difference in the effect of natural COA water is related to the binding of trace minerals found in the water to various proteins in cells, and we are planning a specific study to determine mechanisms. Additional experiments will focus on genetic associations.

## Ethics approval and consent to participate

This article does not contain any studies with human participants or animals performed by any of the authors.

## Authors’ contributions conceptualization and funding acquisition

J.Y.L., J.O.C. and J.J.K. designed and performed most experiments, analyzed data, and prepared the manuscript as a lead author. S.Y.P, N.R., A.S., and V.L. contributed to editing and commenting on the paper. J.J.K., V.L., and S.B.L. supervised the project. This work was supported by grant number E&P-RES20191001-01 to J.J.K and was also supported by grant number E&P-RES20191001-02 to S.B.L. and by the Dorothea Berggren Charitable Foundation.

## Declaration of competing interest

Dr. Lowe is a consultant for AVID Radiopharmaceuticals, Eisai Co. Inc., Bayer Schering Pharma, GE Healthcare, and Merck Research, and receives research support from GE Healthcare, Siemens Molecular Imaging, AVID Radiopharmaceuticals, and NIH (NIA,NCI). Dr. Lee report grants from E&P Co., Ltd. (South Korea) during the conduct of the study.

## Data Availability

The datasets used and/or analyzed during the current study are available from the corresponding author on reasonable request.
