# Supplementary material for: Natural COA water inhibits mitochondrial ROS-mediated apoptosis through Plk3 downregulation under STZ diabetic stress in pancreatic β-cell lines
Source: Biochem Biophys Rep. 2022 Mar 11;30:101247. doi: 10.1016/j.bbrep.2022.101247 (PMC8921297; doi:10.1016/j.bbrep.2022.101247)
Supplement: Multimedia component 1 [file mmc1.pdf]

Biochemical and Biophysical Research Communications

Supplemental data

Analysis of ingredients for COA water

Korea Institute of Geoscience and Mineral resources (KIGAM)

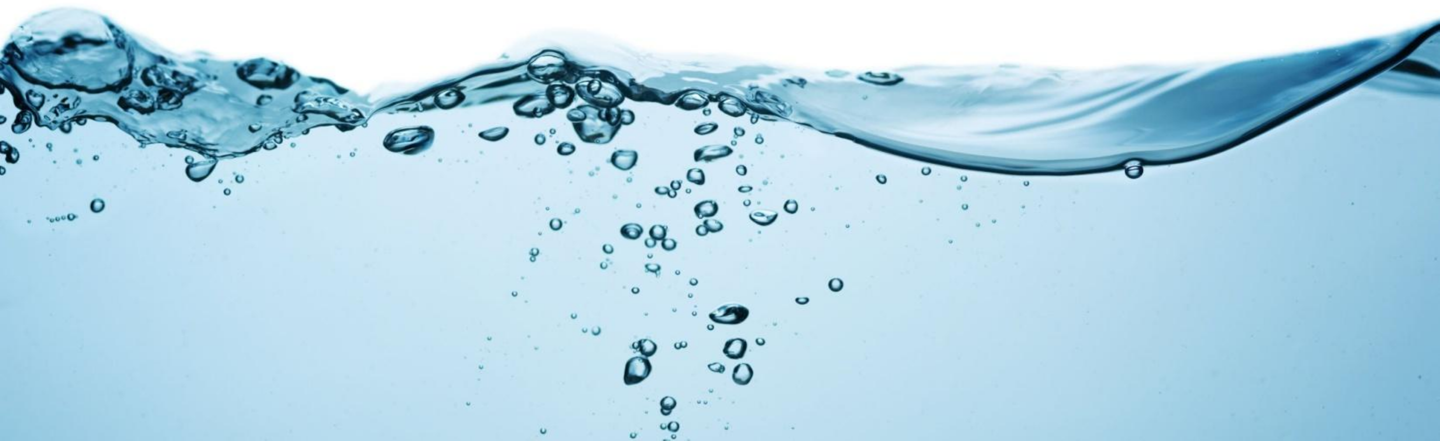

Water Profiles by Manufacturer

	Product Name										
	Evian	Aquafina	FIJI	Icelandic	Evamor	Crystal	COA	Spring	Nestle	Essentia	Ice
**pH**	7.2	6.5-8	7.7	8.4	9	NR	7.5	5.4-7.3	6.5	NR	7.9-8.2
**Br**	NR	NR	NR	NR	NR	NR	<0.01	0-0.014	NR	0.002	0.014-0.02
**Ca**	78	<1.0	18	6.4	1.5	27	16	3.7-12	59	0	45-80
**Cl**	5	1.3	9.8	12	6.4	6	5.4	0-14	18.1	0	1.1-6.6
**F**	NR	0.5	0.25	NR	0.2	NR	0	0-0.25	NR	0	0-0.32
**Mg**	24	20	15	2.4	0	6	9.01	0.67-1.6	10	1.4	16-31
**NO_3**	3.8	<0.50	NR	<0.3	0	NR	2.3	0	NR	0	0
**K**	1	<1.0	4.9	0.6	16	2	0.87	0	1.2	7.7	0-1.3
**Na**	5	3	18	11	66	13	6.3	1.6-9.1	11.9	14	2.5-6.4
**SO_4**	NR	8.6	NR	3.4	NR	36	5	0-8.1	9.7	3.3	10~16
**TDS**	357	NR	222	62	220	NR	NR	0-74	NR	NR	170-310
**HCO3**	357	1.3	155	NR	93	0	NR	NR	184.6	NR	NR
**SIO2**	14	NR	93	NR	NR	NR	25.2	NR	NR	NR	NR

Abbreviations

Potential of hydrogen (pH), Bromine (Br), Calcium (Ca), Chloride (Cl), Fluoride (F), Magnesium (Mg), Bicarbonate (HCO3), Nitrate (NO3), Potassium (K), Sodium (Na), Sulfate (SO4), Total Dissolved Solids (TDS), Slica (SIO2)

Units and Legends

Unit = mg/L, NR = no record
Crystal (Crystal geyser), COA (Dr. COA, natural COA), Spring (Poland spring), Nestle (Nestle pure life), Ice mountain (Ice)

TESTING CERTIFICATE

KIGAM<br>Gwahang-no 124, Yuseong-gu, Daejeon, 305-350, KOREA<br>Tel. +82-42-868-3392, Fax +82-42-868-3393	Registration No. : 120180789A<br>Page(1)/(Total 2)

1. Client
   - ○ Name : Lee Kwang-Wook ○ Organization : COAWATER Co., Ltd
   - ○ Address : 201, 34, Sahwa-ro 18beon-gil, Uichang-gu, Changwon-si, Gyeongsangnam-do, Republic of Korea
   - ○ Date of Receipt : Oct. 11, 2018
   - ○ Arrival Date of Sample : Oct 18, 2018 ○ Date of Payment : Oct. 18, 2018

2. Use of Report : Reference

3. Sample Description : water

4. Date of Test : Oct. 15, 2018 ~ Nov. 01, 2018

5. Test method used : High Resolution ICP-MS

6. Testing Environment
   - ○ Temperature : (23 ± 3) ℃ Humidity : (45 ± 10) % R.H.

7. Test Results

   - Please refer to the attached sheet(s) for details.

Affirmation	Tested by	Technical Manager
	Name Ilyong Park Ilyong	Name YunJung Yoo

The above results are produced by proper procedures based on the National Standard Law §14 and the corresponding retraceable analytical tools.

2018. 11. 30.

Korea Institute of Geoscience and Mineral Resources

- The results included in this test report are characteristics of only the tested sample(s). This Test Report should be used only for the use of report noted in the first page.
- The date of test starts from later date of the arrival date of sample and the date of payment.
- This report should not be used for commercial advertising or as an evidence before court.
- You can ask service dest of Geoanalysis Center of KIGAM(T. +82-42-868-3643) to authenticate this report.
- KIGAM-QP-23-07(back)(2017.09.15.)

TESTING CERTIFICATE

KIGAM
Gwahang-no 124, Yuseong-gu, Daejeon,
305-350, KOREA
Tel. +82-42-868-3392, Fax +82-42-868-3393

Report No. : 120180789A

7. Test Results

(unit : μg/L)

Element \ No.	1
Li	0.98
V	1.26
Cr	0.80
Fe	2.01
Co	0.02
Cu	0.32
Zn	13.5
Mn	0.23
Sr	110
Mo	<0.1
Te	<0.1
Pb	<0.3
Bi	<0.1
Remarks	Dr.COA

• The results included in this test report are characteristics of only the tested sample(s). This Test Report should be used only for the use of report noted in the first page.
• The date of test starts from later date of the arrival date of sample and the date of payment.
• This report should not be used for commercial advertising or as an evidence before court.
• You can ask service dest of Geoanalysis Center of KIGAM(T. +82-42-868-3643) to authenticate this report.
• KIGAM-QP-23-07(back)(2017.09.15.)

TESTING CERTIFICATE

KIGAM<br>Gwahang-no 124, Yuseong-gu, Daejeon, 305-350, KOREA<br>Tel. +82-42-868-3392, Fax +82-42-868-3393	Report No. : 120180789B<br><br>Page(1)/(Total 2)	ilac-MRA KOLAS

1. Client
   ○ Name : Lee Kwang Wook ○ Organization : COAWATER Co.,Ltd
   ○ Address : 201, 34, Sahwa-ro 18beon-gil, Uichang-gu, Changwon-si, Gyeongsangnam-do, Republic of Korea.
   ○ Date of Receipt : 2018. 10. 11 ○ Registration No. : 120180789
   ○ Arrival Date of Sample : 2018. 10. 15 ○ Date of Payment : 2018. 10. 15

2. Use of Report : Reference

3. Sample Description : Water

4. Date of Test : 2018. 10. 15 ~ 2018. 10. 19

5. Test method used : EPA 6010C:2007

6. Testing Environment
   ○ Temperature : (23 ± 2) ℃ Humidity : (50 ± 5) % R.H.

7. Test Results
   • Please refer to the attached sheet(s) for details.
   • This laboratory is not accredited for the test results marked *.

Affirmation	Tested by	Technical Manager
	Name [signature]	Name [signature]

The above testing certificate is the accredited test result by Korea Laboratory Accreditation Scheme, which signed the ILAC-MRA.

2018. 11. 30

Korea Institute of Geoscience and Mineral Resources

Accredited by KOLAS, Republic of KOREA

• The results included in this test report are characteristics of only the tested sample(s). This Test Report should be used only for the use of report noted above.
• The date of test starts from later date of the arrival date of sample and the date of payment.
• This report should not be used for commercial advertising or as an evidence before court.
• You can ask service dest of Geoanalysis Center of KIGAM(T. +82-42-868-3643) to authenticate this report.
• KIGAM-QP-23-06(front)(2017.09.15.)

TESTING CERTIFICATE

KIGAM
Gwahang-no 124, Yuseong-gu, Daejeon, 305-350, KOREA
Tel. +82-42-868-3392, Fax +82-42-868-3393

Report No. : 120180789B

7. Test Results

(unit : mg/L)

Component \ No.	120180789-001
SiO_2	25.2
NOTE	Dr.COA

• The results included in this test report are characteristics of only the tested sample(s). This Test Report should be used only for the use of report noted in the first page.
• The date of test starts from later date of the arrival date of sample and the date of payment.
• This report should not be used for commercial advertising or as an evidence before court.
• You can ask service dest of Geoanalysis Center of KIGAM(T. +82-42-868-3643) to authenticate this report.
• KIGAM-QP-23-06(back)(2017.09.15.)

CERTIFICATE OF ANALYSIS

Issue No.	20010481	Date of Issue	Feb. 04. 2020
Analyzed items	Drinking water(46 Item)	Analytical Purpose	For Periodic inspection
Applicant	Company Name	E&P Co.,Ltd	
	Address	130-53, Jiseon-ro, Oesan-myeon, Buyeo-gun, Chungcheongnam-do, Republic of Korea	
Date of Application	Jan. 31. 2020		

No.	ITEM	STANDARD	RESULT	No.	ITEM	STANDARD	RESULT
1	General bacteria	100 CFU/ml AND LESS	0	24	Benzene	0.01mg/L AND LESS	N · D
2	Total Colony Counts	N· D/100mL	N · D	25	Toluene	0.7mg/L AND LESS	0.002
3	Fecal Coliforms/ E. Coli	N· D/100mL	N · D	26	Ethylbenzene	0.3mg/L AND LESS	N · D
4	Pb	0.01mg/L AND LESS	N · D	27	Xylene	0.5mg/L AND LESS	N · D
5	F	1.5mg/L AND LESS	N · D	28	1,1-Dichloroethylene	0.03mg/L AND LESS	N · D
6	As	0.01mg/L AND LESS	N · D	29	Carbonte tetrachloride	0.002mg/L AND LESS	N · D
7	Se	0.01mg/L AND LESS	N · D	30	1,2-Dibromo-3-Chloropropane	0.003mg/L AND LESS	N · D
8	Hg	0.001mg/L AND LESS	N · D	31	1,4-dioxane	0.05mg/L AND LESS	N · D
9	CN	0.01mg/L AND LESS	N · D	32	Hardness	1,000mg/L AND LESS	100
10	Cr	0.05mg/L AND LESS	N · D	33	Consumption of $KMnO_4$	10mg/L AND LESS	0.7
11	NH_3-N	0.5mg/L AND LESS	N · D	34	Odor	ODERLESS Except for the disinfection odor	N · D
12	NO_3-N	10mg/L AND LESS	1.5	35	Taste	TASTELESS Except for the disinfection taste	N · D
13	Cd	0.005mg/L AND LESS	N · D	36	Cu	1mg/L AND LESS	0.017
14	B	1.0mg/L AND LESS	N · D	37	Color	5 AND LESS	1
15	Phenol	0.005mg/L AND LESS	N · D	38	ABS	0.5mg/L AND LESS	N · D
16	Diazinon	0.02mg/L AND LESS	N · D	39	pH	5.8-8.5	6.8
17	Parathion	0.06mg/L AND LESS	N · D	40	Zn	3mg/L AND LESS	0.030
18	Fenitrothion	0.04mg/L AND LESS	N · D	41	Cl^-	250mg/L AND LESS	5.9
19	Carbaryl	0.07mg/L AND LESS	N · D	42	Fe	0.3mg/L AND LESS	N · D
20	1,1,1-Trichloroethane	0.1mg/L AND LESS	N · D	43	Mn	0.3mg/L AND LESS	N · D
21	Tetrachloroethylen	0.01mg/L AND LESS	N · D	44	Turbidity	1NTU AND LESS	0.30
22	Trichloroethylen	0.03mg/L AND LESS	N · D	45	SO_4^{-2}	200mg/L AND LESS	11
23	Dichloromethane	0.02mg/L AND LESS	N · D	46	Al	0.2mg/L AND LESS	N · D

Result	Pass
Standard Excess Item	

We here by certify that the above are correct.

Hanwool Life Science Inc. PRESIDENT

26-18, Gajeongbuk-ro, Yuseong-Gu, Daejeon, Republic of Korea

This certificate is only for the product which is sampled by the applicant and can not be used for commercial advertisement without pre-approval.
